# The MAPS: Toward a Novel Mobility Assistance System for Visually Impaired People

**DOI:** 10.3390/s22093316

**Published:** 2022-04-26

**Authors:** Katerine Romeo, Edwige Pissaloux, Simon L. Gay, Ngoc-Tan Truong, Lilia Djoussouf

**Affiliations:** 1 LITIS Lab, University of Rouen Normandy, 76800 St-Etienne-du-Rouvray, France; edwige.pissaloux@univ-rouen.fr (E.P.); ngoc-tan.truong@univ-rouen.fr (N.-T.T.); lilia.djoussouf@univ-rouen.fr (L.D.); 2 LCIS Lab, University of Grenoble Alpes, 26000 Valence, France; simon.gay@univ-grenoble-alpes.fr

**Keywords:** visually impaired mobility assistance, tactile tablet, tactibelt, maps’ learning, space awareness emergence, path planning, wayfinding

## Abstract

This paper introduces the design of a novel indoor and outdoor mobility assistance system for visually impaired people. This system is named the MAPS (Mobility Assistance Path Planning and orientation in Space), and it is based on the theoretical frameworks of mobility and spatial cognition. Its originality comes from the assistance of two main functions of navigation: locomotion and wayfinding. Locomotion involves the ability to avoid obstacles, while wayfinding involves the orientation in space and ad hoc path planning in an (unknown) environment. The MAPS architecture proposes a new low-cost system for indoor–outdoor cognitive mobility assistance, relying on two cooperating hardware feedbacks: the Force Feedback Tablet (F2T) and the TactiBelt. F2T is an electromechanical tablet using haptic effects that allow the exploration of images and maps. It is used to assist with maps’ learning, space awareness emergence, path planning, wayfinding and effective journey completion. It helps a VIP construct a mental map of their environment. TactiBelt is a vibrotactile belt providing active support for the path integration strategy while navigating; it assists the VIP localize the nearest obstacles in real-time and provides the ego-directions to reach the destination. Technology used for acquiring the information about the surrounding space is based on vision (cameras) and is defined with the localization on a map. The preliminary evaluations of the MAPS focused on the interaction with the environment and on feedback from the users (blindfolded participants) to confirm its effectiveness in a simulated environment (a labyrinth). Those lead-users easily interpreted the system’s provided data that they considered relevant for effective independent navigation.

## 1. Introduction

**Figure 1:**
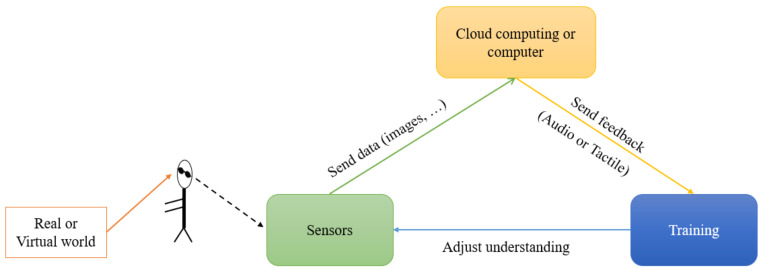
Sensory substitution loop.

Autonomous navigation in an unknown environment is one of the greatest challenges for a VIP as vision plays an important role in gathering the information necessary for many processes involved in this complex task. In the last decade, many research projects were developed to compensate for the loss of vision, most of them relying on sensory substitution. Sensory substitution is grounded in the idea of replacing an impaired or lost sense with another sense [1]. Paul Bach-y-Rita, pioneer in this field, aimed to work at restoring visual functions in blind people [2]. The usual sensory substitution devices (SSDs) aspire to efficiently convey visual data in real-time via touch or hearing. This data may include the shape and/or size of an object, the perceived (ego-centered) distance from it, or the color of the object [1,3]. Typical SSDs consist of the following three components: a sensor, a processing unit that simplifies and converts the sensory information, and a user interface to transmit this information to the user. All SSDs are based on the sensory substitution motor loop (cf. Figure 1).

This loop presents the embodiment of perceptions: (1) The sensor (usually a camera) is pointed in a given (ego-centered) direction (to the target). (2) A cloud computing or a computer interprets the image and converts it to tactile or audio stimulations. Then the user receives and interprets these stimulations (audio and tactile descriptions), and the brain generates the ad hoc percept. (3) During training, the user tests percepts while interacting with the space via the received feedback. Through iterations of the sensory-motor loop, the VIP adjusts understanding of the code to match perceptions with the sensory feedback that was perceived.

Presently, some SSDs cannot transfer the volume and complexity of visual information with the precision and speed suitable to fit the vision-based task. They lack spatial and temporal resolution and also bandwidth [4]. Schinazi et al. [5] presented the topic of functional reorganization of perceptual modalities considering new developments for SSDs based both on locomotion and wayfinding. Consequently, some SSDs try to understand how some specific elements improve and assist navigation and wayfinding [6,7,8,9].

Navigation usually involves both wayfinding and locomotion tasks [10]. Locomotion is closely linked to the ability to localize obstacles and negotiate a path around them, while wayfinding involves the orientation in space and ad hoc path planning in any environment (large environments included). Both tasks are easier to implement having a visual input [11,12,13,14]. However, locomotion and wayfinding involve different components of decision making, different skills [10], and require different characteristics of visual information. For example, in locomotion tasks, vision is used to update distance information to an obstacle [12,13]; in wayfinding tasks, vision helps in spotting points of interest for mobility (PIM), landmarks, cues, and clues useful for navigation guidance. Consequently, SSDs should be geared to answer the specific demands of both locomotion and wayfinding to convey the specific information needed for both tasks. Therefore, to efficiently assist the navigation, we need to develop a novel system that supports both locomotion and wayfinding, thus allowing the emergence of spatial awareness; the proposed system is named the MAPS.

The paper is organized as follows: Section 2 outlines the state of the art on SSDs, while Section 3 presents a novel model of VIP mobility and overviews the TactiBelt and F2T designs, the two components of the MAPS system. Section 4 presents the TactiBelt detailed design (for its potential reproducibility). Section 5 outlines some preliminary evaluations of the TactiBelt with VIP and blindfolded persons which confirm relevance of MAPS for the target assistance. Finally, Section 6 summarizes our ideas and discusses future developments of the MAPS system

## 2. State of the Art on SSDs

Over the years, several researchers have approached the substitution of the visual sense using the hearing or tactile senses [14,15]. For visual-to-audio SSDs, two of the most popular devices are “the vOICe” [16,17,18] and “EyeMusic” [19].

The vOICe converts gray-level visual images by scanning them in video mode (from left to right, from top to bottom). Each pixel is converted into a sound, based on its luminance and the pixel’s orthogonal coordinates in the image. High luminance pixels present the sound louder than low luminance pixels. The pixels on the left of the visual field are played before those on the right, and pixels at the top have a higher pitch than those at the bottom [20]. The vOICe allows VIP individuals to access visual information through hearing to recognize and localize the object after a long training [3].

‘EyeMusic’ transforms the entire scene visual parameters (shape, location, brightness, and color) into sound. It uses different instrumental sounds to perceive brightness and color.

However, the interpretation of the output signals of these devices is difficult and requires long training phases to understand the represented scene [21,22,23]. Space awareness is difficult to acquire. Moreover, for navigational tasks, the constantly changing perspective and distance while moving cannot be processed in real-time. A VIP has difficulty differentiating multiple objects, especially those vertically aligned, as they have difficulty distinguishing between the pitches of the sounds that are played simultaneously. Furthermore, such devices cover environmental audio cues.

To overcome these limits, tactile-visual sensory substitution systems were proposed. The Brainport (or TDU, Tongue Display Unit) is one of the most popular SSD devices. This device transforms visual images into a pattern of electrical stimulations delivered via an electrode array that is placed on the tongue [1,24]. The users explore tactile patterns representing a scene by using this electrode pad. Therefore, objects can be processed theoretically in parallel [25], and they do not have difficulty distinguishing between vertically aligned objects.

With decades of research, despite ambitious aspirations and impressive achievements, few devices have been accepted by the VIP in their daily life, and no one device has become widespread as none effectively improve the life quality of the VIP [4,21,26]. Chebat et al. [27], identified several drawbacks of the current forms of SSDs and proposed some promising approaches that attempt to circumvent them. These are: learning, standardization of training, temporal coherence, reduction of the cognitive load, orientation, depth, contrast, assisted functions and costs and dissemination; they are shortly described in this paper.

*The learning problem*: With the current SSDs, the end users need a lot of time for practice and training [7,8,24]. Learning skills with a new SSD that contradict received mobility training could impair previously acquired mobility skills and discourage potential users from using SSDs.

*The standardization of training*: In this field, many publications examine certain SSD elements, but each paper has a new protocol to fit its needs of methodology. The performance of SSD devices are difficult to compare due to the lack of standardization. Optimizing the learning processes and standardizing the performance would assist the perceptual training and the guidance of potential users through steps needed to interpret the information provided by a device. This would solve *the learning problem*.

*The temporal coherence*: For an SSD to be useful in navigation, the image of the user’s surroundings needs to be presented and interpreted in real-time for a user’s possible immediate processing. Some SSDs are designed based on audio which transfers the visual information into sounds using the temporal flow. That can add a small delay in the delivery of the 2D message to the user [28]. On the other hand, some SSDs are designed based on touch, such as the TDU [7,8], which can transmit the visual information in real-time. However, their interpretation is sometimes slow due to cognitive load induced by the complexity of the tactile images.

*The cognitive load*: This problem is directly linked to the complexity of the algorithms used to generate substituting stimuli, which ultimately need to be learned by the user. The more complex the interpretation of SSD information, the more difficult the completion of the sensorimotor loop presented in Figure 1. Therefore, the simultaneous interpretation of the information provided by the SSD and accomplishment of a task requires important cognitive burden. Finding the balance between minimal and necessary information which should be provided by the SSD is fundamental.

*The orientation*: This problem is closely related to the accurate (precise) localization of objects in space using SSDs. The direction information provided by the SSD is often confusing, and although participants can detect objects in the field of the sensor’s activity, they often report being unable to tell exactly where the sensor points in the environment. To localize an object in space accurately, the depth of the viewed scene should be as constant as possible, and the relevant feedback must be provided. Proper training in remapping must be optimal to achieve the appropriate distal attribution of the moving stimulus.

*The depth problem*: It is difficult to detect the distance to the obstacles, and avoid them [7] if depth information is lacking. However, some recent devices can calculate depth information. For example, with Eyecane the end-users can understand the depth information through vibrations and sounds [22].

*The contrast problem*: Many SSDs can work well under optimal contrast conditions; however, under different conditions or with any other settings, they may not work correctly, such as the TDU.

*The resolution problem*: Downsampling of the image resolution resolves issues to use another modality, but it reduces the resolution of data. That makes it harder to recognize the details of a scene. Nevertheless, zooming in can improve this problem, for example EyeMusic [29].

*The cost problem*: The cost of SSDs is still high because of the long research and development phases. Some companies and laboratories can reduce the cost of SSDs by developing their prototypes on the existing devices (ex. Smartphones). However, the high price is still a problem to accept and provide to end users.

*The dissemination problem*: Many scientific journals are not always easily accessible to the VIP, especially the 2D data such as graphs and figures. We should disseminate the results of scientific research to all, including the VIP.

Although some attempts have been made to overcome the above listed problems, they still have some limits. For example, the Eyecane is easy to use and requires little training but has a low resolution. The vOICe and the EyeMusic offer a higher resolution, but they comprise complex coding that makes them more difficult to use and they, consequently, require many hours of training. Therefore, we propose the MAPS, a novel system for VIP mobility assistance based on the journey approach implementation learned in mobility classes. It offers a good compromise between conveying high-level information for navigation, data resolution, and its usability. It uses two hardware cooperating devices, the F2T, a tactile tablet for electronic (imaged) map accessibility based of the force–feedback principle, and TactiBelt, a haptic belt providing real-time information on nearest obstacles and on target to reach.

## 3. The MAPS, a Novel System for VIP Mobility Assistance

**Figure 2:**
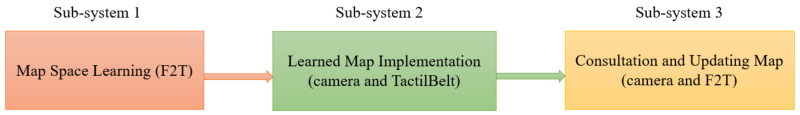
Model of VIP mobility assistance.

The MAPS system for VIP mobility assistance consists of three subsystems as shown in Figure 2. Subsystem 1 assists the “Map space learning” using the tactile tablet F2T (Force Feedback Tablet). The goal of this subsystem is to help the VIP memorize the map of the environment where they will move. After preparing the journey, the VIP starts it (using the white cane) and may benefit from the assistance provided by Subsystem 2: a shift from “learned (memorized) map” into physical navigation using TactiBelt, its accessories (such as a camera) and associated software (space perception control and journey control via the mobility graph—a kind of VIP specific GPS). By providing the mobility graph built on a map supporting the path integration navigation strategy, Subsystem 2 aims to help the VIP move more independently and lower stress and cognitive load. During the journey, if the users forget the map memorized information, they can use the information provided by Feedback 3, which is a “consultation and updating map” displayed on the F2T. The goal of this feedback is to help the VIP recall the map of its nearest space. Feedback 3, a specific software running on the F2T, works similarly to the classic GPS (and is still in development).

The subsequent subsections provide overviews of the MAPS Subsystems 1 and 2.

### 3.1. Feedback 1: Map Space Learning

**Figure 3:**
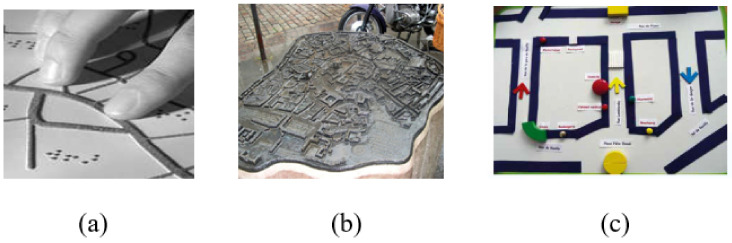
Map learning: (**a**) thermoformed map; (**b**) concrete map; (**c**) magnet-based map.

Today, map information has different media: thermoformed maps, concrete maps, and magnet-based maps as shown in Figure 3. However, such media have their drawbacks: their display is static and at a fixed scale, they have a fixed predefined (north-south) map orientation, and their content is difficult to exploit during the journey.

**Figure 4:**
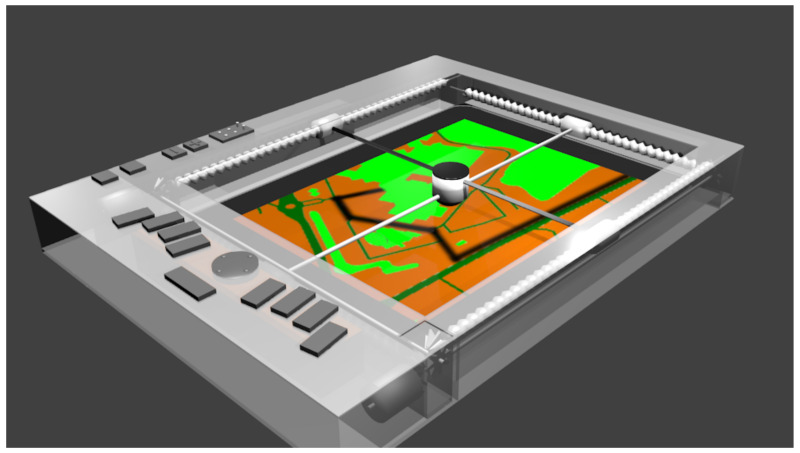
F2T model design (with the simplified map of Rouen Normandy University).

**Figure 5:**
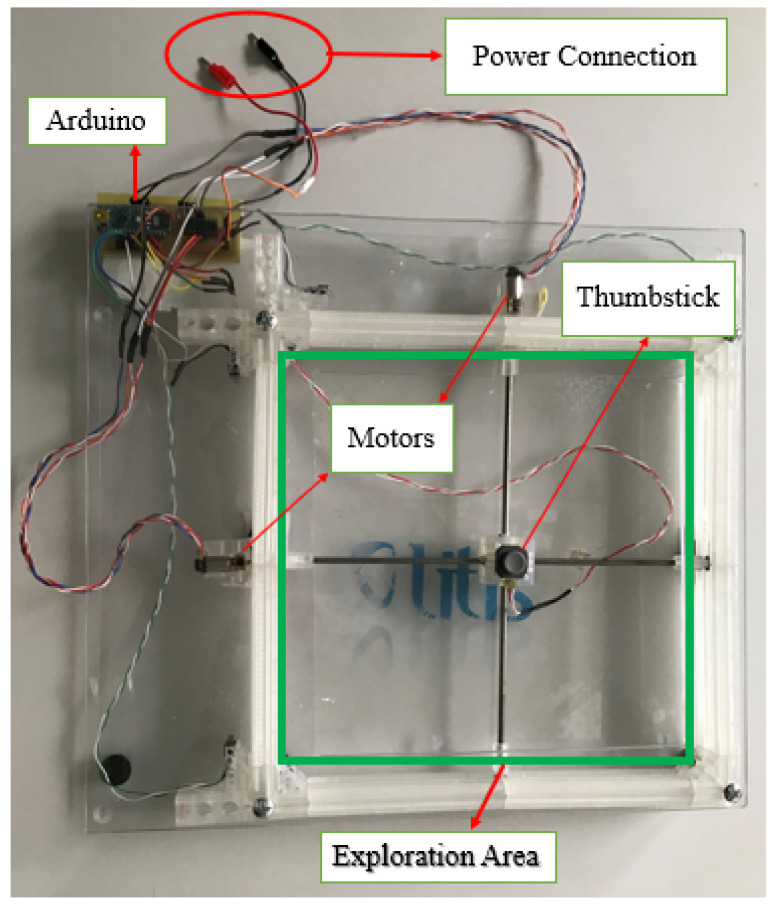
F2T current prototype.

To overcome these limits, we propose an interactive tactile tablet based on the force–feedback principle, hence its name F2T, force–feedback tablet (cf. a model design on Figure 4 and a current prototype on Figure 5). Our current prototype is activated by two small gear motors moving a thumb stick controlled by an Arduino Nano board (ATmega328 microcontroller) communicating with a PC through a USB and a graphical interface dedicated to haptic environment development and test, developed in Java. Detailed design and prototyping of the F2T are provided in [30].

The general scenario of “map space learning” can be summarized as follows:

(1) The user selects the area to explore through audio commands and F2T buttons.

(2) The map is loaded from a GIS (geographic information system) provider and automatically converted into its equivalent topological representation. The proposed journey path is also provided, (cf. Figure 4 black line on the simplified map of Faculty of Rouen Normandy University).

(3) The Points of Interest for Mobility (PIM), useful to both confirm journey progress and lower independent mobility stress are added to the uploaded map (map annotation).

(4) Known PoIs (points of interest in the usual sense) are uploaded from GIS (roads, fountains, building, shops,…) and converted into localized sound sources of the audio of the MAPS system. This audio-enhanced journey path is accessed through the F2T which allows the user to explore the map with the use of a thumb stick/joystick (controlled by a force feedback mechanism).

**Figure 6:**
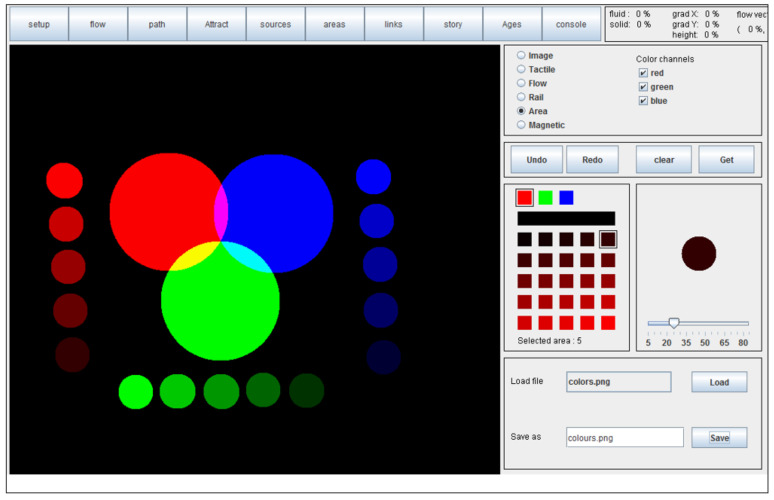
Examples of color-coded representations of the haptic effects used to simulate image properties. Red channel corresponds to fluid friction, blue channel to solid friction, and green channel to the elevation of the shape.

The F2T provides the graphic content of images’ spatial information by 2D force feedback. The displayed information can be explored by moving a mobile thumb stick whose movements’ resistance levels vary depending on the basic information (e.g., slowing down or stopping the user when trying to move over a wall). The F2T can provide passive effects (textures and reliefs), active effects (dynamic scene), and actively guided movements during the exploration. Passive and active feedback is used to convey information about the map (space organization) during a *free exploration*, while active guidance is used to provide *direct guidance along a path*. Examples of simple “tactile images” can be seen in Figure 6, where colors represent different types of frictions used for feedback generation.

We divide the passive feedback into two basic categories based on the user’s actions with respect to the functional map:

- Friction feedback: The F2T can simulate both solid and fluid friction, allowing different textures to be presented.
- Elevation feedback: This effect can be used to simulate slopes and bas-relief elements. A high elevation difference also allows edge simulations, making it possible to follow the shape of an object.

Furthermore, we can create more complex tactile paths by combining passive and active feedback. For example, we make “canyons” where the user is oriented to exit from either side. If the user tries to push his/her finger toward other directions, the force feedback will simulate a slope to push the end user’s finger to the canyon bottom. This canyon indicates the “walkable” paths or areas that allow the user to only move in some directions.

### 3.2. Feedback 2: Effective Displacement Using TactiBelt

**Figure 7:**
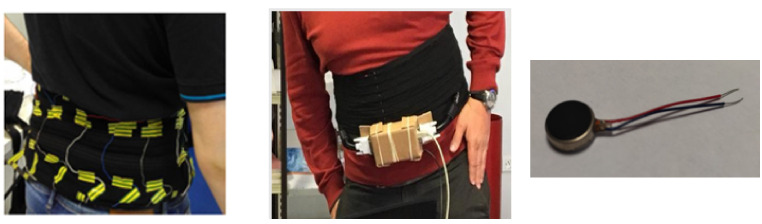
TactiBelt prototype (**left**, showing vibrator positions, and **center**, showing the complete device) and type of used vibrator (**right**, RS DC minivibration motor).

The memorized map is the basis for effective displacement with a cane via our original TactiBelt (Figure 7). We designed a new prototype based on the recommendations of SSDs. The TactiBelt is designed with vibrator motors and is worn around the waist. This kind of interface is discreet, can be worn under a large pullover, and allows the end users to perceive ego-centered spatial information. The belt has three layers of vibrators to encode different information on distal obstacles (surface located (cane detectable obstacles and over a distance of up to 5 m) and overhanging obstacles (the upper row)). This prototype will add two front-facing cameras that are embedded into a pair of glasses. They are then combined with an inertial unit to provide depth information about nearby obstacles. A GPS/Galileo chip will provide absolute localization and ego-centered distance information about nearby landmarks. Cartographic data will be collected from online services or from buildings’ blueprints for indoor navigation. However, the first prototype (only TactiBelt) will be tested in a virtual environment (Section 5).

**Figure 8:**
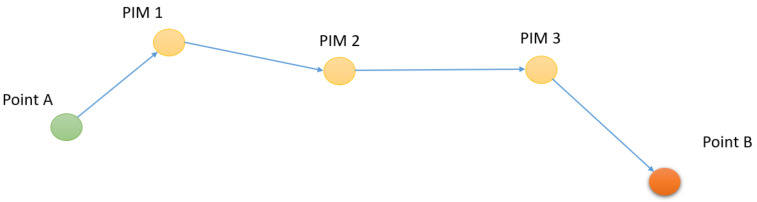
Movement strategy from point A to point B through Points of Interest for Mobility.

Some prototypes are designed using a vibrotactile system [31,32,33] or a commercially available Sunu Band (https://www.sunu.com/, accessed on 23 April 2022), to enhance the peripheral visual detection of the VIP. They transfer only the information of obstacles to a vibration motor (distance, orientation, elevation). In addition to providing information on obstacles, the TactiBelt can assist during physical (or virtual) displacement via movement from point A to point B by a set of intermediate steps performed along the adjacent segments, each segment linking two consecutive PIMs (Figure 8). The practical implementation of this strategy is based on a mobility graph, extracted from the annotated geographic map [34]. The physical displacement between adjacent nodes is supposed to be performed straightforward. The path integration algorithm is based on our bio-inspired indoor and outdoor mobility model [35].

**Figure 9:**
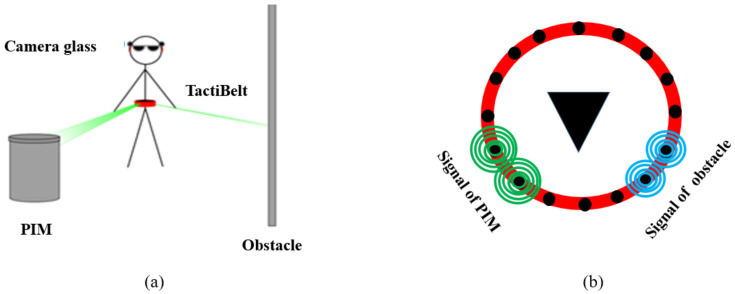
TactiBelt combined with glasses (stereo rig): (**a**) working principle; (**b**) generated stimulations. Green circles represent the next PIM to reach with a discontinuous vibration pattern. Blue circles represent the nearest obstacles with a continuous vibration pattern.

While moving, thanks to vibrators, the TactiBelt provides to the VIP two types of information on the 3D environment, virtual or real (cf. Figure 9): the nearest obstacle (blue circles) and the next PIM of the mobility graph (green circles). A specific vibration indicates the final journey PIM (“target is reached”). The position of the activated vibrator indicates the ego-direction of the obstacle/PIM, while the amplitude of vibrations indicates the distance to obstacles/PIM (knowing that the vibration amplitude is inversely proportional to the distance). The continuous vibration pattern is used for nearest obstacle information, and the discontinuous vibration pattern is used for the next PIM to reach. Section 4 will present the TactiBelt hardware design.

## 4. TactiBelt Hardware Design

From a hardware point of view, the TactiBelt consists of a belt made of elastic fabrics with 46 miniature vibrators (cf. Figure 7 right). It is driven by a unique microcontroller (Arduino Mega, ATMega2560) and powered by an external “power bank” type battery. The intervibrator distances are uniform at the waist which correspond to recent physiological findings [36].

### 4.1. The TactiBelt Operative Part

The Arduino board is equipped with a custom shield developed to power vibrators with an external battery. The control board is placed in a box in the front of the belt and is connected to the belt (and thus to the vibrators) with two DVI cables to facilitate maintenance of the device. These cables have 24 connected wires, allowing for a connection of 46 vibrators (a wire is used for common VCC), although the shield can control up to 48 vibrators.

**Figure 10:**
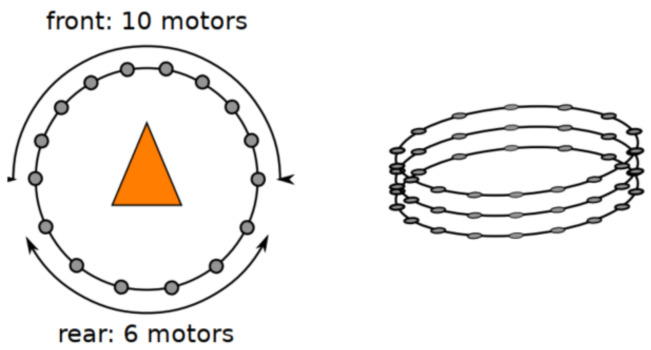
Vibrator distribution on the belt (the triangle provides the orientation of the user’s gaze).

The belt has 46 vibrators distributed as follows: three rows of vibrators going around the user’s waist (Figure 7 and Figure 10). On the rows, the vibrators are spatially equidistant which matches the known distribution of the human waistline mechanoreceptors. The two upper rows have 16 vibrators, while the lower row has 14 vibrators. The current distribution foresees on each row, 10 vibrators at the front and 6 or 4 vibrators at the back. Indeed, the front part must allow a better discretization of the space (thus better obstacle detection). The use of three rows allows the belt to localize the obstacles located above the walking surface, at the chest level and difficult to detect with a cane (cf. Section 3.2). The TactiBelt control system is presented in Section 4.2.

### 4.2. The TactiBelt Control Part

The management of the vibrators is entirely performed by the Arduino board. The vibrators can be controlled individually with the amplitude of the vibrations, the period, and the width of the pulses. The microcontroller can also send a predefined number of pulses to transmit a particular code.

Each vibrator is designated by an identifier (“spatial coordinates” on the belt). A vibration is identified with four parameters:

- The power “*p*”, characterizing the amplitude of the vibrations, controlled with a high-frequency PWM;
- The duration “$t_{1}$”, corresponding to the duration at the high state of the pulses;
- The duration “$t_{2}$”, characterizing the duration at the low state of the pulsations. Note that if $t_{1}$ = 0 or $t_{2}$ = 0, the vibration will be continuous;
- The parameter “*n*”, specifying the number of pulses; if *n* = 0, the pulsation will not stop.

The vibration power is defined by pulse width modulation (PWM). The pulse period is 8.4 ms. Note that this maximum power corresponds to 50 % of the maximum power of the vibrators. This limitation makes it possible to avoid discomfort related to significant vibrations while reducing the consumption of the device.

The durations $t_{1}$ and $t_{2}$ allow the definition of a pulsation. If $t_{1}$ or $t_{2}$ is null, the vibration will be continuous. The pulsation has a period $t_{1}+t_{1}$, with a high state of duration $t_{1}$ and a low state of duration $t_{2}$.

**Figure 11:**
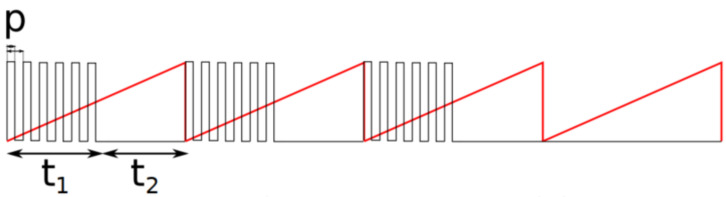
Signal of a vibrator. On this schema, PWM and pulse periods are not represented with the same scale. The signal corresponds to the parameters *p* = 45%, $t_{1}$ = 0.3 s, $t_{2}$ = 0.3 s, *n* = 3. The vibrator stops after 3 pulses (*n* = 3). The red signal is the signal carrying period 0.6 s.

The vibrator signal data are provided in Figure 11. The parameter “*n*” allows the specification of a finite number of pulses (1 to 9 pulses). The vibrator stops after the number of pulses specified by *n*. If *n* = 0, the signal will not be interrupted.

## 5. Experimental Evaluation of TactiBelt

Our system consists of two devices: F2T and TactiBelt. The evaluation of the F2T was presented in [30]. Collected results indicate that the F2T can be used to convey graphical information to blind users through force–feedback. This paper presents the preliminary evaluation of TactiBelt only.

The first experiments using TactiBelt were organized in two phases:

(1) Strength of the stimuli and perception of direction (Section 5.1);

(2) Navigation in a simulated environment (a serious game) (Section 5.2).

These experiments involved seven blindfolded participants (three women and four men), grouped into two age groups: below 30 years old (four participants) and above 30 years old (three participants). This last subdivision is suggested by the user’s experience related to the usage of haptic/tactile technologies. Therefore, gender and age are two variables in our experiences, and the collected results will be analyzed using them. Table 1 gives the ages of the seven participants to our tests. The age of the participants varies from 22 to 68 with an average of 35.85.

### 5.1. Perception of Direction and Strength of the Stimuli

The goal of these tests is twofold: (1) check the technical quality of generated stimuli (Section 5.1.1), and (2) learn the mapping (spatial perception) (Section 5.1.2).

#### 5.1.1. Check the Technical Quality of Generated Stimuli

The evaluation of the quality of tactile stimuli generated by vibrators confirms the technical specifications of the vibrators (amplitude and frequency) and allows selecting the amplitude of the vibrations the most suitable for each participant for the subsequent experiments (part of the user profile for the MAPS system).

#### 5.1.2. Perception of Direction

##### Task

During the learning of the mapping, the participants tested the tactile stimuli of the TactiBelt vibrators by a pointing task–indication of a 3D point ego-direction, the source of their tactile stimulation.

##### Experimental Platform

**Figure 12:**
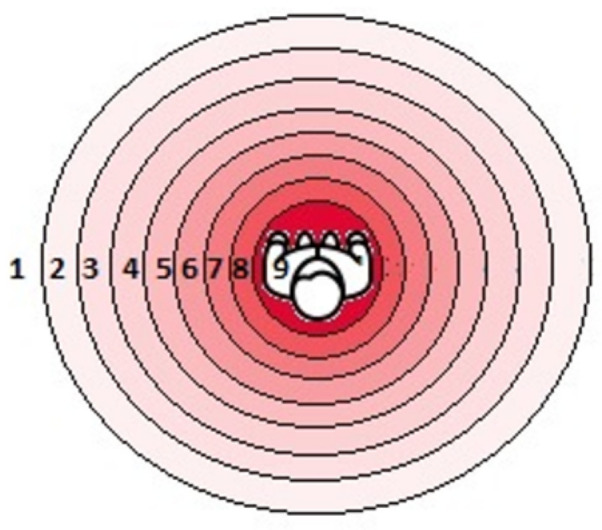
Test pattern for direction perception with a TactiBelt (calibration chart): the colored circles represent the space around the user. When moving the computer mouse on the circles, the vibrators are activated; a vibrator characterizes the ego-orientation and ego-distance of the pointed 3D spot. Each circle corresponds to a precise amplitude of the vibration, from the greatest (level 9) to the lowest (level 1).

This task used an ego-directional calibration map representing a set of nine concentric circles of growing radii (cf. Figure 12). The vibrators were activated more or less strongly to simulate the orientation and distance of a 3D point (supposed to be the source of vibrating stimuli). The concentric circles define the distance from the user and therefore the power of the considered activated vibrator (from one to nine). Each circle represents the strength of the stimuli, the closest circles to the center create the most powerful stimuli to represent the obstacle or the target that is near the person. The outer circles create less powerful stimuli to show the gradual, inversely proportional, distance of the obstacle or the target.

##### Experimental Protocol

The experience leader moved the computer mouse between the circles. A vibrator was powered more or less strongly and generated stronger or weaker tactile stimulation on the TactiBelt worn by the participant. The position of the vibrator indicated the potential ego-direction of the 3D elements in the space (e.g., an obstacle). Two kinds of information were expected to be given by a participant:

(a) To point on the TactiBelt, the spot where the generated tactile stimulation was perceived, and indicate the direction of the potential 3D point which induced this stimulation;

(b) To assess its growing or lessening power of the stimuli (distance estimation to a 3D point).

##### Collected Data

The blindfolded participants proved an accurate perception of the 3D ego-direction of the tactile stimuli. The interpretation of the stimuli was intuitive and effortless; the reaction to the stimuli was very rapid.

The perception of stimuli power (cf. Figure 12) was tested by all the participants with the ascending and descending staircase procedure. A vibration was perceived very lightly with the ascending method at level two and confirmed by all the participants at level three. With the descending method, all participants confirmed the perception of the vibrations until level three. The differences in vibration power levels were observed for levels 9-7, 7-5, and 5-3. A difference of the minimum two levels were perceived by all. This last observation is useful to interpret the distance to the obstacle while moving an avatar in the simulated environment (cf. Section 5.2)

##### Discussion

From the collected data, it could be deduced that there are no differences linked to the gender or the age. This confirms that the stimulations are well perceived by any person and means that the TactiBelt may be effectively accepted by everybody showing that it is an inclusive device.

### 5.2. Navigation in the Simulated (Virtual) Environment

#### Task

The goal of this experiment—a serious game— was to test the efficiency of the TactiBelt to provide along a path without/with an obstacle data on the obstacle and to allow space awareness emergence. The tested hypothesis claims that TactiBelt assists the navigation toward a goal (final PIM) by providing vibration data as an indicator of the information (PIM, obstacles) for a VIP and blindfolded participants.

#### Experimental Platform

**Figure 13:**
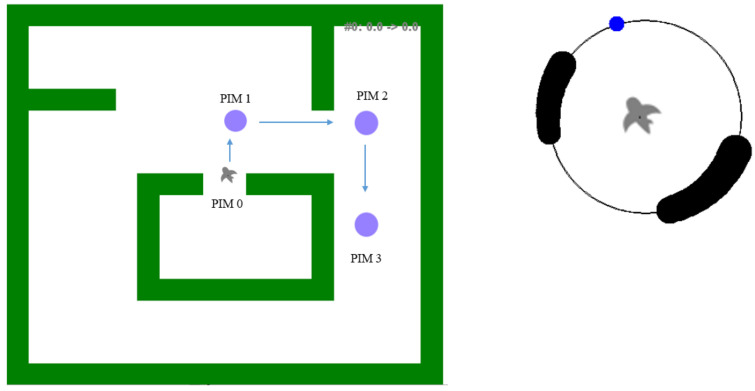
Simulated maze. On the **left**: the initial position of the avatar (“a bird”) in the labyrinth. The next targets (PIM1, PIM2, and PIM 3) to reach are represented by a violet circle. PIM 3 is the destination. On the **right**: the representation of the environment on the TactiBelt matching the avatar’s initial position: in black, the presence of nearby obstacles; in blue, the direction of the target to reach (colors represent different patterns of vibrations); the avatar is in the TactiBelt center.

The second test was performed in a simulated environment (a maze). This simulation environment allowed testing the belt with several types of information simultaneously: the presence of obstacles (e.g., walls), the directions of the target, etc. This serious game used a simulated environment, a kind of labyrinth (cf. Figure 13 left).

The environment perceived by the avatar’s vision system (cf. Figure 13), thus by the avatar, was simulated. it used a polar (ego-)reference frame and generated (via the power of the signal) the distance to obstacles within the field of view of 360° and angular resolution of 1°. This information was used to localize the TactiBelt vibrator and its vibration level (which encodes the distance).

#### Experimental Protocol

**Figure 14:**
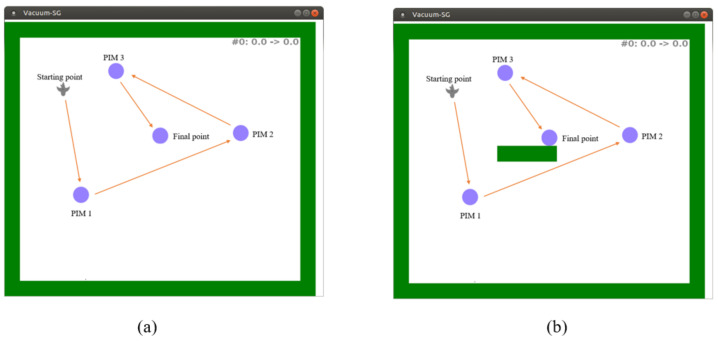
Simulated environment: (**a**) without obstacle; (**b**) with an obstacle between the avatar and the target (the blue circle).

The experiment had two levels of difficulty: (1) Without obstacle (Figure 14a), to validate the hypothesis of the navigation possibility toward a goal using TactiBelt stimulations; (2) With an obstacle between the avatar and the target (Figure 14b) to validate that the vibrations of the PIM are distinguishable from the vibrations of obstacles. This experiment aimed to confirm the movement strategy from point A to point B through PIMs (Figure 8). This test had four paths: Path 1 was from starting point to PIM1, Path 2 was from PIM1 to PIM2, Path 3 was from PIM2 to PIM3, andthe final path was from PIM 3 to the final point (Figure 14); the positions of the starting point, PIMs, and the final point were the same for two levels.

**Figure 15:**
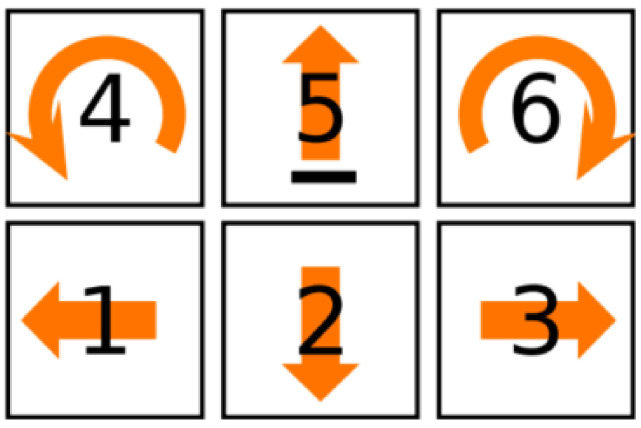
Mapping of the keys of the numeric keypad to direct the avatar. This key mapping was selected as num-5 key usually has an ergot making it easier to recognize.

Only tactile information generated by the TactiBelt could be used to navigate in the environment. This tactile information was built from vision data provided by the (simulated) vision system. The tactile information was translated into a physical displacement of an avatar using a PC numeric keypad as shown in Figure 15. Key 4 was to rotate left, Key 5 was to go forward, Key 6 was to rotate right, Key 2 was to go back, and Key 1 and Key 3 were to go to the left and to the right, respectively. All these operations were performed in avatar ego-centered reference frame.

**Figure 16:**
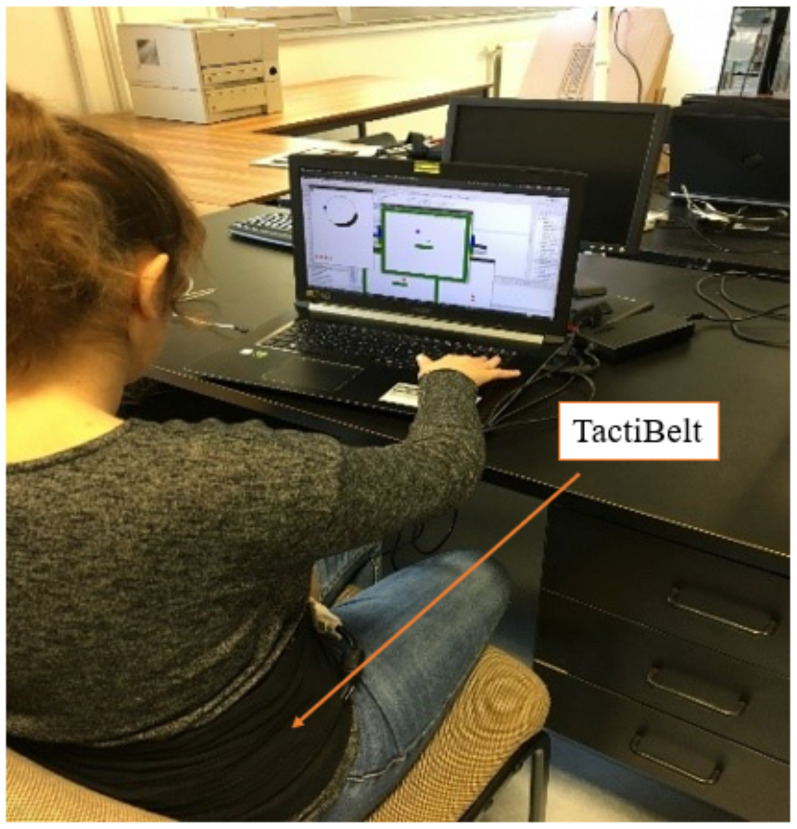
Navigation of a VIP in a simulated environment using the TactiBelt.

Our experiment was preceded by a training. It let the participants familiarize themselves with TactiBelt before starting the experiments, as learning rates differ between participants [37]. During the learning stage, blindfolded participants could try the TactiBelt in the simulated environment to get familiarized with the numeric keypad to move the avatar and with the target signal which is a regular repeating signal easy to identify (Figure 16).

#### Collected Data

**Figure 17:**
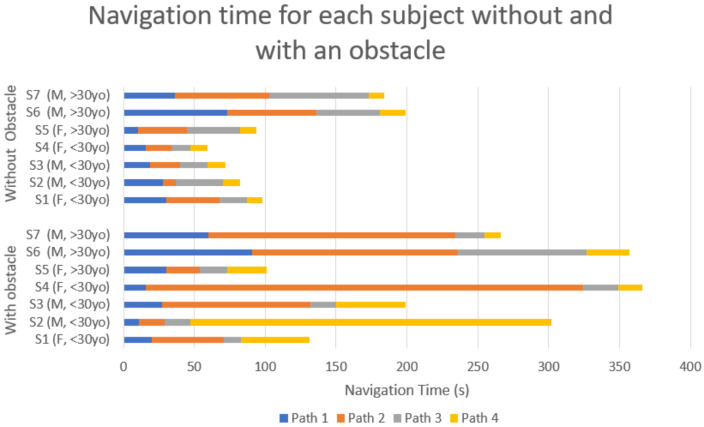
Navigation times from starting point to final point of each subject with and without an obstacle for four paths.

Figure 17 presents the detailed time of each participant to complete the four paths, reaching successively targets PIM1, PIM2, PIM3, and the final point. Overall, the participants had no difficulties moving the avatar toward the PIMs without the obstacle. With the obstacle, most participants (five of seven participants) took more time on Path 2 (from PIM1 to PIM2). This can be explained by the presence of an obstacle on this path.

**Figure 18:**
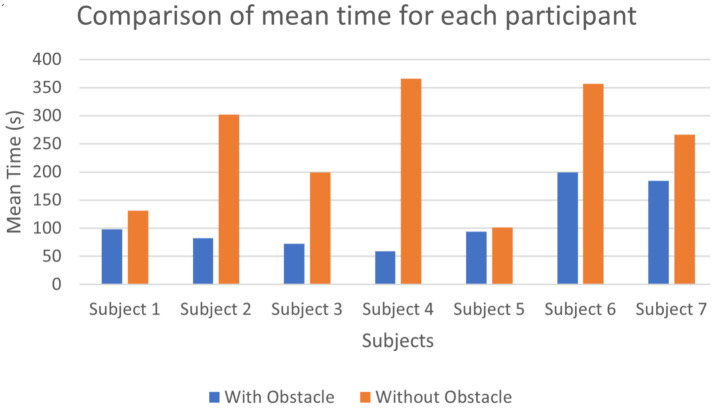
Mean time to touch the target PIM of each participant without (blue) and with an obstacle (red).

To complete the data from Figure 17 and Figure 18 shows the mean time of each participant with and without an obstacle. The overall average time to touch the target PIM without an obstacle was 28.1 s (SD = 13.9 s), and the average time with an obstacle was 61.5 s (SD = 26.4 s).

#### Discussion

For the tests with an obstacle, participants had to differentiate the vibrations of the obstacle from the repeating signal of the target. The participants said that the signal of the obstacle was perceived very distinctly as the signal was continuous and was very different from the signal of the target (PIM). When the avatar was getting closer to the obstacle, the vibrations could be felt more and more powerfully, and the participant needed more time to move the avatar.

Sometimes, the signal of the obstacle could be in the same direction as the target. In this case if the participant was close to the obstacle, the signal of the obstacle was more powerful than the signal of the target. Therefore, the participants found a procedure to make a distinction between an obstacle and the (final) PIM: they moved the avatar around the obstacle to perceive more powerfully the target and to approach it.

The two age groups (less than 30 years old, and more than 30 years old) were chosen according to the use of tactile and touch stimulation technologies in Figure 17 and Figure 18. We observed that younger participants reacted quicker than the older participants to handle the numeric keypad due to their use of video games. Women performed slightly better than men for the task without obstacles. This last observation confirms the results obtained by other authors [38]. Future studies with a larger group of participants will provide data on the effect of age and gender due to trends noted in our data.

The obtained results with navigation to a target in the absence of an obstacle show that previous usage of tactile/touch stimulation technologies (thus some habit to interpret the tactile stimuli) positively impacts the speed of tactile navigation. This point is encouraging data for our MAPS system’s future appropriation by the VIP. However, as seen in Figure 17 and Figure 18, overall younger participants found as much difficulties as older participants in the presence of an obstacle. It is to be noted that the learning process of the navigation system only involved tasks without obstacles.

The tests were applied only once to see the usability and perception of the TactiBelt stimulations. This is a limitation since repeated trials would have provided data that could determine if there were learning effects beyond the initial practice period. Future works will analyze learning effects with and without obstacles.

#### Conclusion of Two Experiences

Collected data validated the proposed architecture of the TactiBelt and its hardware implementation. The information provided by TactiBelt can be correctly interpreted and allowed the participants to navigate toward the target (in the absence and the presence of an obstacle) in a simulated environment.

Moreover, this test helps us answer some problems that were presented in Section 2. First, the information about the learning and training phase are important to improve our training protocol which can make the use of our system easier. In addition, the users felt the feedback (audio, tactile) with no noticeable delay and could react instantly (solution to the temporal coherence). In the first prototype, we tested our system with a virtual environment and found that our algorithm works efficiently with no delay and no extra cognitive load (solution to the cognitive load problem). In the near future, we will test it in a real environment with our cameras and evaluate this problem. Concerning the orientation problem, our system provides the direction information of nearest obstacle and next PIM. The users confirmed that they quickly recognized this information (solution to the orientation problem).

Furthermore, we are completing our system which includes two front-facing cameras combined with an inertial unit to provide stable orientation-aware depth information about nearby obstacles. A GPS/Galileo chip will be added to provide absolute localization and ego-centered distance information about nearby landmarks (next PIM). Based on that, cartographic data will be collected from online services. This design will solve the depth, the contrast, and the resolution problems. In addition, our system can help a VIP access independently open source literature (article, 2D graphics …). Then they can give us feedback to improve our system (solution to the dissemination problem).

## 6. Conclusions

Autonomous navigation is one of the biggest challenges for a VIP. This paper introduces a new system for the assistance of the VIPs’ mobility.The MAPS is composed of two original digital subsystems: F2T-TactiBelt. The originality of the proposed approach comes from the MAPS ability to assist the VIP’s real-time displacements. This system assists different subtasks of the mobility process and is especially useful for target reaching, namely:

- To learn a map and thus to construct the mental map of the environment where the VIP will navigate (using F2T);
- To transfer the “learned map” into a physical displacement (using the TactiBelt and its accessories).

The preliminary results of the experimental evaluation of the TactiBelt with the VIP and blindfolded participants in a simulated environment show that TactiBelt provides relevant data for secure and independent moving toward a target in a static environment. The provided data can be easily interpreted by the VIP, which signifies the probable acceptance of the MAPS.

Future work will focus on improving the MAPS systems with more reliable hardware and software. The spatial distribution of TactiBelt vibrators should be precisely investigated using touch senses physiology. The F2T should be designed as a frame to be clipped on classic PC screens, which will be used as the control of a MAPS system and lead to a truly portable device. The simulated stereo apparatus, a part of Feedback 2, must be replaced by a “real vision system”, a stereo apparatus embedded in a pair of glasses, and associated with an inertial measurement unit (IMU) (for various obstacles detections and for balance sense simulation). This system will be enhanced with a GPS (or a Galileo) chip for efficient outdoor tracking and reinforcement of our bio-inspired indoor and outdoor mobility model [35]. Cartographic data necessary for navigation in real environments (indoor and outdoor) will be collected from online services or building blueprints for indoor navigation.

We will also investigate the use of audio effects to generate interactive multimodal representations of the map. Finally, additional serious games should be designed with more complex topologies than the considered virtual environment and corresponding to real configurations while navigating indoors and outdoors.

New tests will be carried out to measure the difference between a controlled virtual environment with and without typical distractions. To this effect, in the future we want to add nonstatic obstacles and other types of distraction that can occur outdoors to test our prototype. We will extend our testing population to elderly subjects.

Our first evaluations involved navigation in a virtual world, but it is important to note that since the proposed prototype is to be used in a real outdoor situation, it will be necessary to conduct the evaluation of the system, with the addition of other sensors, to determine preliminary efficacy of the prototype with the lead users, the VIP.

It is also to be noted that the first evaluation of the prototype involved blindfolded sighted people, conducted in a simulated environment. As such, it does not reflect the possible performance of actual VIPs. This is why we are getting in contact with some charities with VIPs to conduct next evaluations.

## Figures and Tables

**Table 1:** The gender and age of the participants of our tests.

Subjects	Gender	Age
1	F	24
2	M	22
3	M	25
4	F	24
5	F	68
6	M	48
7	M	40

## Data Availability

The data presented in this study are available on request from Edwige Pissaloux, project leader (Edwige.Pissaloux@univ-rouen.fr). The data are not publicly available due to privacy issues.

## Abbreviations

The following abbreviations are used in this manuscript:

VIP	Visually Impaired People
MAPS	Mobility Assistance Path Planning and orientation in Space
SSDs	Sensory Substitution Devices
PIM	Points of Interest for Mobility
TDU	Tongue Display Unit
F2T	Force Feedback Tablet
PWM	Pulse Width Modulation
